# Treatment of Conjunctivitis Neonatorum

**Published:** 1870-07

**Authors:** E. L. Holmes

**Affiliations:** Professor of Ophthalmology and Otology, in Rush Medical College, and Surgeon to the Chicago Charitable Eye and Ear Infirmary


ARTICLE III.—*Treatment of Conjunctivitis Neonatorum.* By E. L. HOLMES, M. D., Professor of Ophthalmology and Otology, in Rush Medical College, and Surgeon to the Chicago Charitable Eye and Ear Infirmary.

(Read before the Illinois State Medical Society.)

There are two methods of treating conjunctivitis of new-born infants, each recommended more or less urgently by different authorities.

By one plan, very weak solutions of the ordinary astringents are injected or instilled under the lids at short intervals.

By the second plan, a much stronger caustic astringent is applied to the palpebral conjunctiva—the portion especially affected —once, or, in unusually severe cases, twice daily.

Not wishing to dwell upon the nature and causes of the disease, I will simply state that it is, without doubt, in by far the larger portion of cases, simply a catarrhal conjunctivitis.

Infection, either from leucorrhœal or, especially, gonorrhœal discharge in the vagina of the mother, must be regarded as a less frequent cause than many have supposed.

A certain diagnosis in reference to the cause of the disease, is almost absolutely impossible, in consequence of the very great tendency of simple catarrhal conjunctivitis in infants to assume strictly a purulent form.

The treatment, therefore, must be based upon the character and amount of the discharge from the eyes, rather than upon the cause of the inflammation.

This inflammation, if not early arrested, is exceedingly liable to become in the highest degree purulent. This marked tendency to purulent discharge, seems to depend upon some condition of the conjunctiva peculiar to infancy; perhaps no more readily explained than the fact that the iris in infancy is less susceptible than that of adults.

The point, to which I ask especial attention at this time, is in reference to the difficulty of ensuring a suitable application of mild remedies in violent forms of inflammation.

It is undoubtedly true that so long as there is a little swelling, and the discharge contains scarcely any muco-purulent matter, the frequent application of weak solutions may be entrusted to the nurse.

However competent this method may be even in severe cases, it must be remembered that frequency and thoroughness of the applications are the conditions on which success depends.

The fear, on the part of mothers and nurses, of causing pain and crying, in very many, if not in almost all, instances, prevents the thorough application of these remedies. It is well known that the simple act of holding the head and opening the eyes causes an infant to cry violently.

Physicians in the country and usually those in the city are unable to visit an infant more than once or twice daily. In addition to the inefficient manner in which the remedies are too often employed, there is danger of injury to the eye in introducing the point of the syringe. Moreover, there is danger not altogether small that the stream from the syringe may accidentally carry purulent matter from the patient to the eye of the attendant.

For the past eight or ten years I have invariably employed in severe cases the second plan I mentioned, which consists in making an application, once or at most twice daily, of a strong caustic astringent.

In nearly all the cases which I am called to treat, either in my own practice or in consultation, the disease has continued a week or more with great swelling and discharge.

The application is made in the following manner: after the palpebral conjunctiva has been as extensively exposed as possible, and thoroughly cleansed from moisture by gently pressing upon the inflamed surface a soft piece of linen, quite a large camel's hair brush, merely moistened with a solution of argent. nit., gr. x.–xx. to the ounce, is lightly passed over the conjunctiva.

Great precaution should be taken that the solution is applied to as much of the conjunctiva as possible, and in such a manner that it shall not flow over the cornea. The eyes should be gently cleansed almost every hour with tepid water.

Ulceration or even sloughing of the cornea do not contraindicate the use of the applications above described.

It should be remembered, however, that the indiscriminate use of strong caustics instilled between the lids, cannot be too earnestly deprecated.

The obstetrician, undoubtedly, meets with more of these cases in the early stages than the oculist, and relieves many patients by the very mildest means, when they are thoroughly applied by experienced nurses. The number of such cases, however, which are finally referred to the oculist, must be an evidence that such treatment is very often either in itself inefficient, or almost of necessity improperly applied.

It is not the object of this paper to discuss the treatment necessary in serious complications, but simply to remove from the mind of the general practitioner all fear regarding the use of strong caustic solutions (argent. nit.) in infantile conjunctivitis, provided they are applied in minute quantities equally distributed over the whole surface.

